# Supplementary material for: Decoupling species richness and interaction frequency reveals how fungal interactions regulate wood decomposition
Source: ISME Commun. 2026 Sep 17;6(1):ycag267. doi: 10.1093/ismeco/ycag267 (PMC13625648; doi:10.1093/ismeco/ycag267)
Supplement: Supplementary file 1 [file supplementary_material_ycag267.zip › Chiba_suppleExperiment_ycag267.pdf]

Decoupling species richness and interaction frequency reveals how fungal interactions regulate wood decomposition

Supplementary experiment: Competition on agar medium

Methods

Prior to the wood block microcosm experiment, the competitive abilities of the four fungal strains were tested on 2% MA arena in Petri dishes with 9 cm diameter. The amount of MA in each dish was 10 mL. The inoculum agar plugs were cut out from the actively growing mycelium on 2% MA using a sterilized cork borer (5 mm diameter). The plugs of different strains were placed on a MA arena at a distance of 3 cm apart from each other. To create a contact of the two strains at the center of the dish, the slow-growing strains were inoculated several days earlier to the fast-growing strains.

The dishes were incubated at 25°C in the dark for 2–4 weeks until one of the two competitors in the dish completely replaced its counterpart (R) or the two competitors in a dish made a zone line at the interaction front and deadlocked (D). The competition of the four strains was settled as a full-factorial design (a total of six combinations), and three replicates were prepared for each combination. To compare the competitive abilities of the four strains, a score of 1 was given to the winner in R dishes, –1 to the loser in R dishes, and 0 to each strain in D dishes. The scores of all dishes were summed up to make the total score of each strain.

Results

The competition outcomes of the four fungal species on 2% MA are shown in Table S2 and Fig. S1. *Og*, *Tv*, and *Fk* were deadlocked to one another. *Fk* was also deadlocked with *At*. *Og* and *Tv* replaced *At*. All three replicate dishes showed the same outcome in all competition. Based on these results, the competition scores were 3 for *Og* and *Tv*, 0 for *Fk*, and −6 for *At*.
