# Supplementary material for: Decoupling species richness and interaction frequency reveals how fungal interactions regulate wood decomposition
Source: ISME Commun. 2026 Sep 17;6(1):ycag267. doi: 10.1093/ismeco/ycag267 (PMC13625648; doi:10.1093/ismeco/ycag267)
Supplement: Supplementary file 1 [file supplementary_material_ycag267.zip › Chiba_suppleFigs_v4_ycag267.pdf]

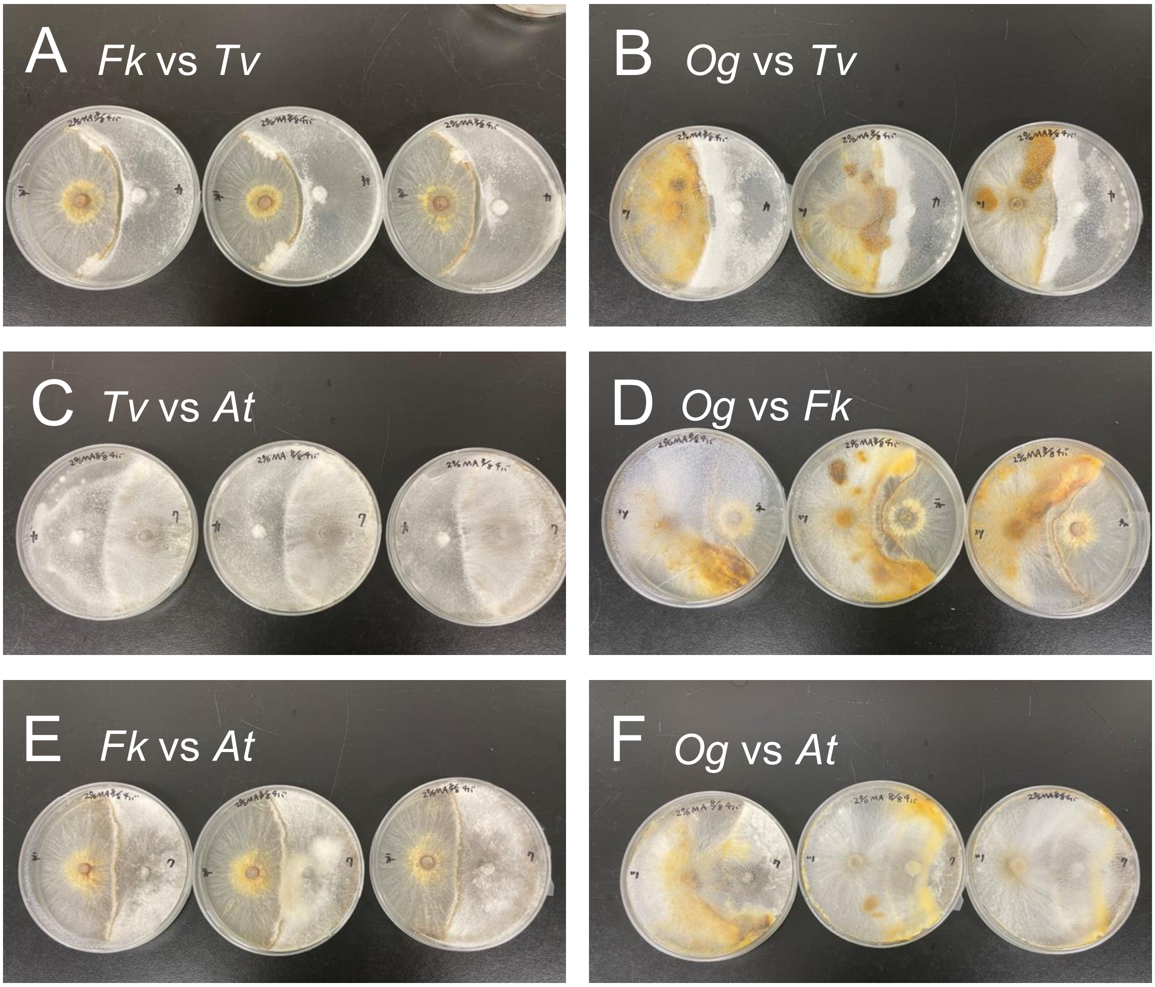

Fig. S1 Competition outcome of the four fungal species on 2% Malt extract agar media after incubated at 25°C in the dark for 2–4 weeks. Each photo shows the results of the three replicate dishes. The three replicates showed identical results in all combinations. (A) *Fk* (left) versus *Tv* (right), deadlocked. (B) *Og* (left) versus *Tv* (right), deadlocked. (C) *Tv* (left) replaced *At* (right). (D) *Og* (left) versus *Fk* (right), deadlocked. (E) *Fk* (left) versus *At* (right), deadlocked. (F) *Og* (left) replaced *At* (right).

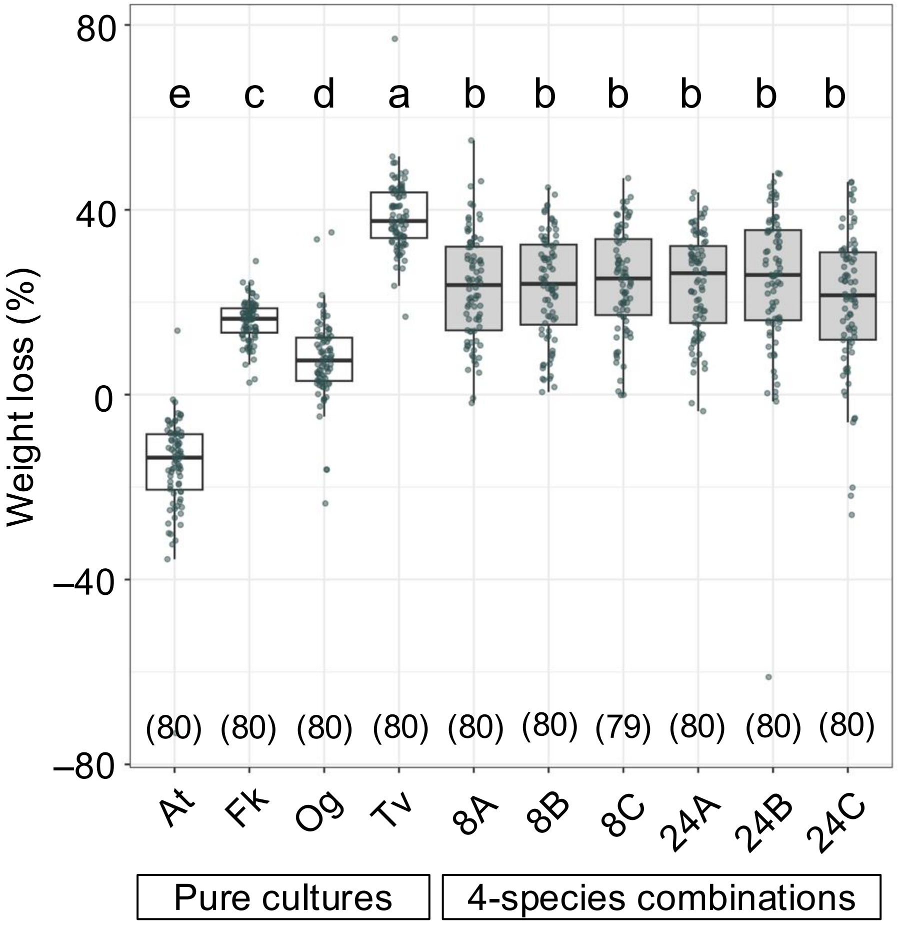

Fig. S2 Weight loss of the wood blocks in four-species experiments. The four white boxes at the left show the results of pure cultures, and the six gray boxes at the right show the results of mixed cultures. See Fig. 1 and 2 for the species locations in the mixed cultures. The different letters shown above each box indicate significant difference (Steel–Dwass test, $p < 0.05$). The numbers enclosed in parentheses are the sample number.

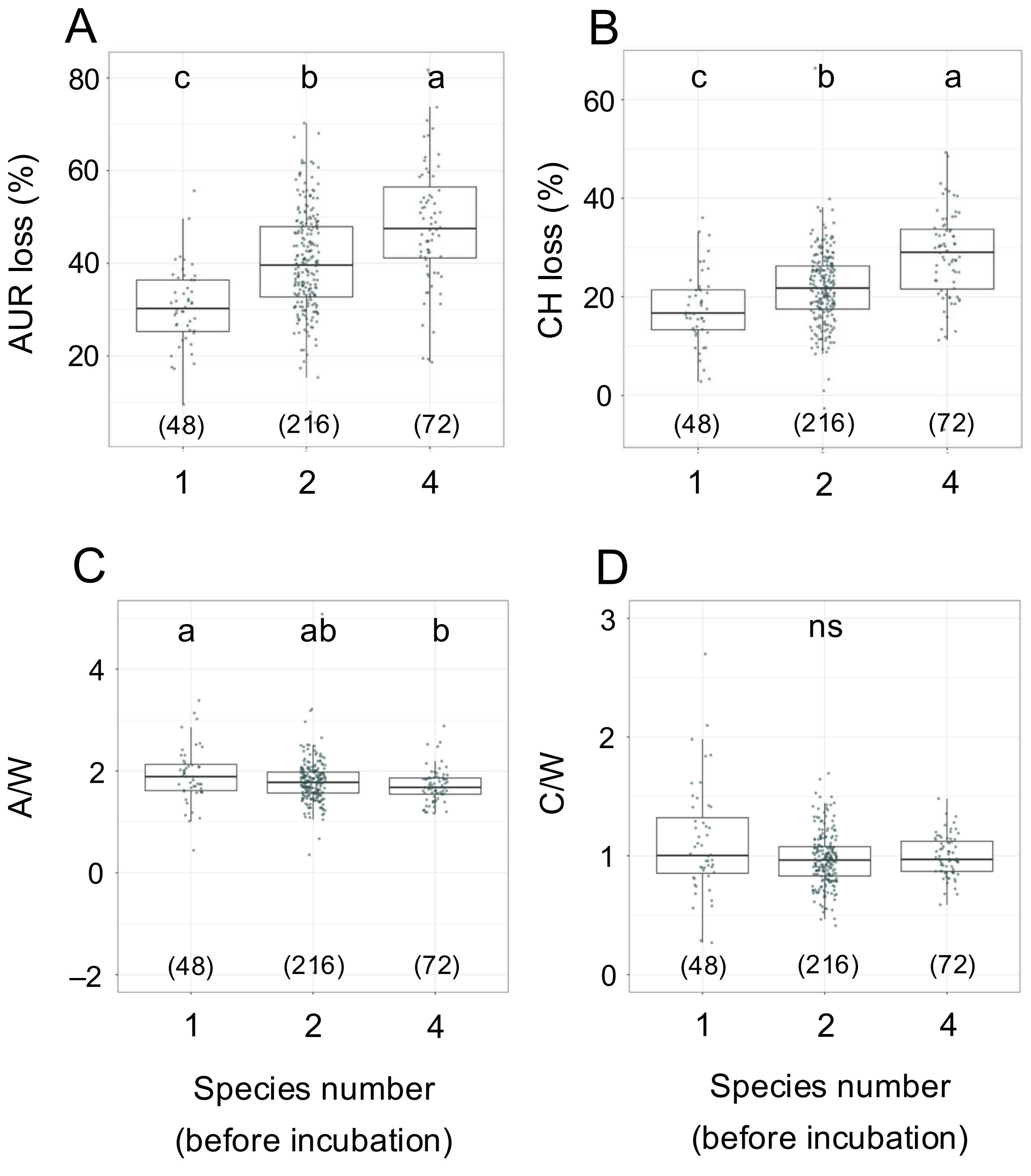

Fig. S3 Effects of fungal species number before incubation on the weight loss (%) of AUR (A) and carbohydrate (B) in *Fk* blocks, and the relative weight loss of AUR (C) and carbohydrate (D) against weight loss of wood. The data of 336 *Fk* blocks without replacement and zone lines were used. Two outliers in each A/W and C/W were out of the plots. The different lowercase letters indicate significant difference across the blocks with different fungal species number (Steel–Dwass test, $p < 0.05$). ns, not significantly different. The numbers enclosed in parentheses are the sample number.

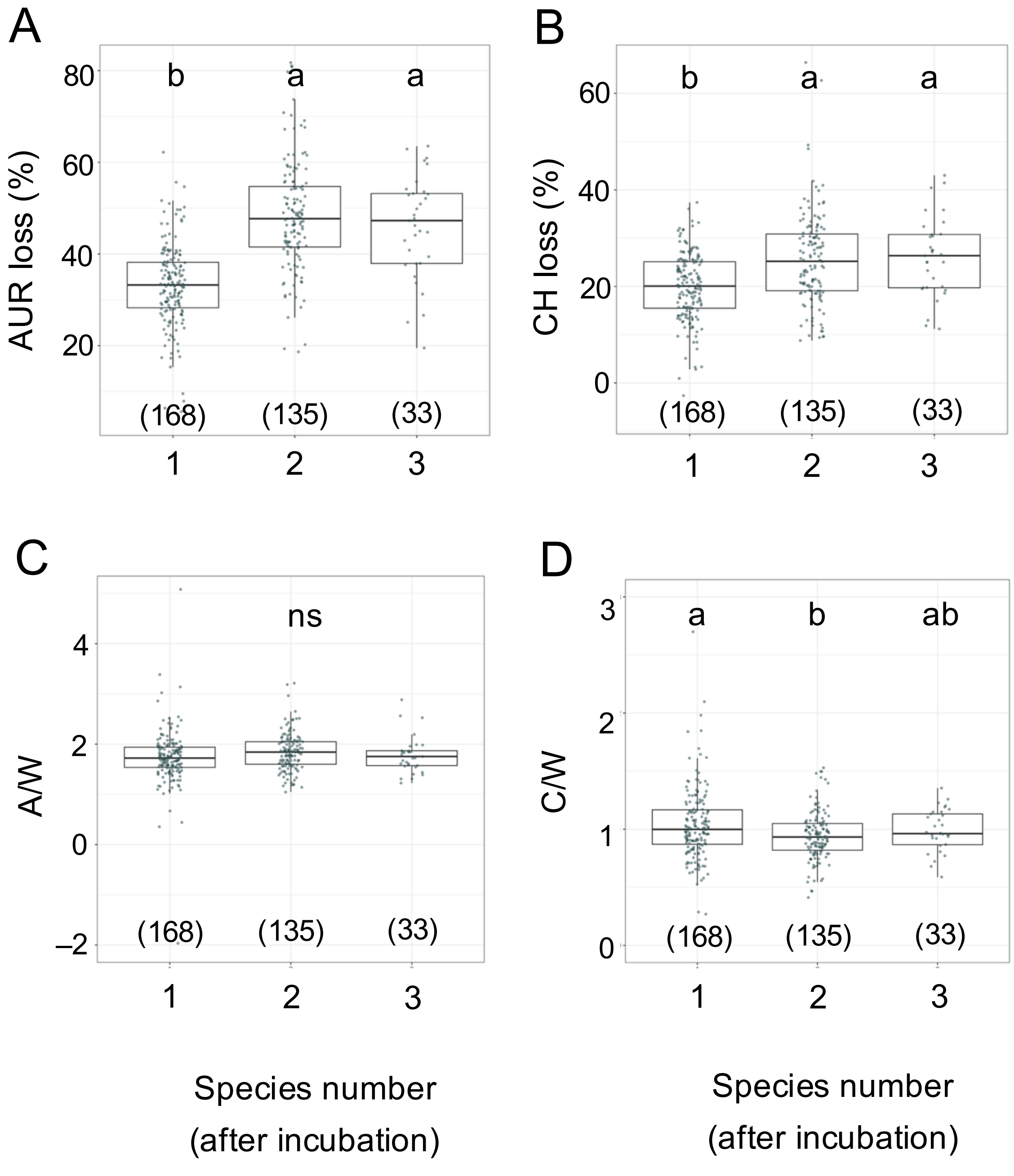

Fig. S4 Effects of fungal species number after the incubation on *Fk* blocks' weight loss (%) of AUR (A) and carbohydrate (B), and relative weight loss of AUR (C) and carbohydrate (D) against weight loss of wood. Data of 336 *Fk* blocks without replacement and zone lines were used. Two outliers in each A/W and C/W were out of the plots. The different lowercase letters indicate significant difference across the blocks with different fungal species number (Steel–Dwass test, $p < 0.05$). ns, no significant difference. Note that none of the four-species experiments retained original four species after the incubation. The numbers enclosed in parentheses are the sample number.

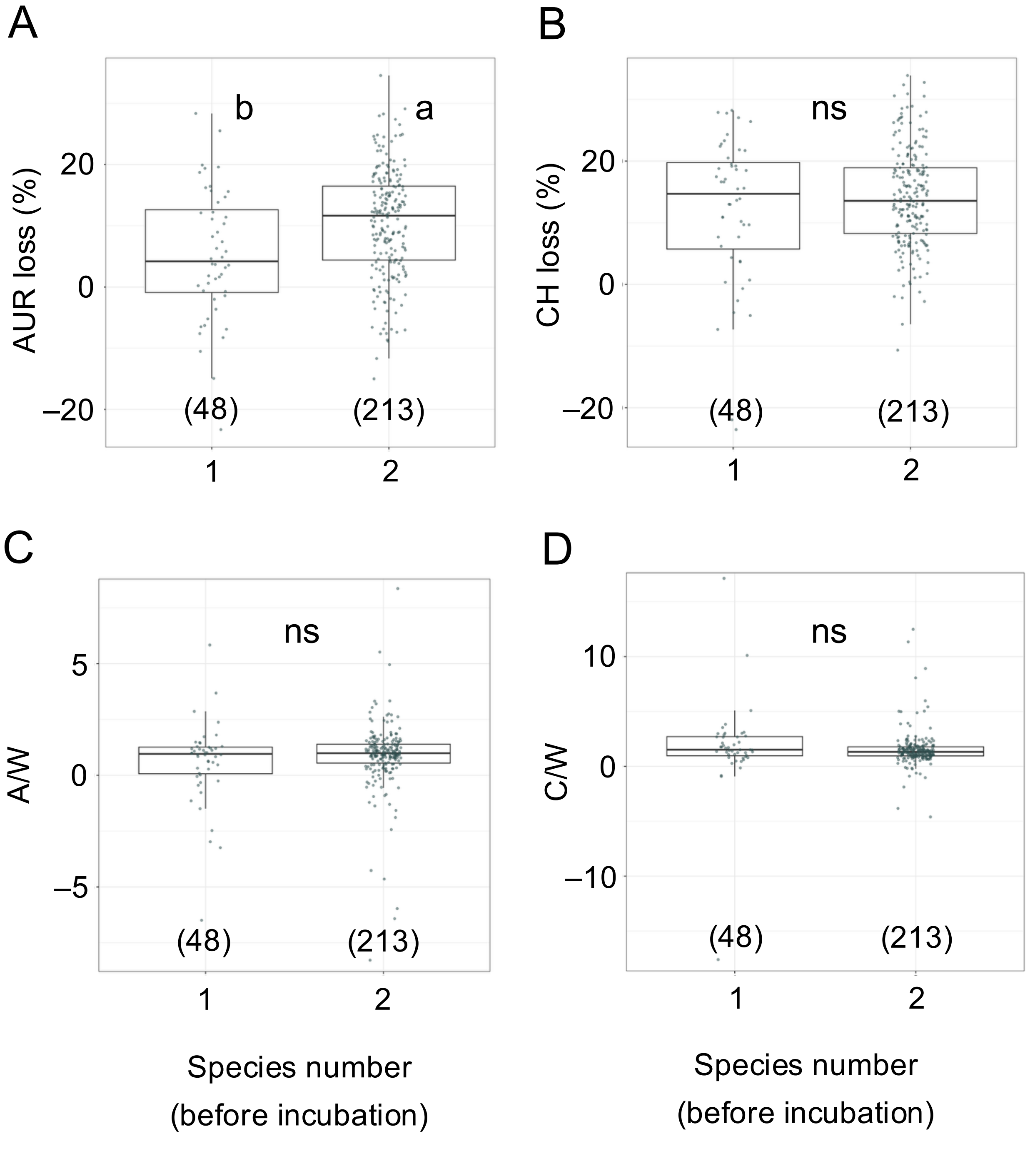

Fig. S5 Effects of fungal species number before incubation on the weight loss (%) of AUR (A) and carbohydrate (B) in *Og* blocks, and the relative weight loss of AUR (C) and carbohydrate (D) against weight loss of wood. The data of 261 *Og* blocks without replacement and zone lines were used. Four outliers in each A/W and C/W were out of the plots. The different lowercase letters indicate significant difference across the blocks with different fungal species number (Steel–Dwass test, $p < 0.05$). The numbers enclosed in parentheses are the sample number. ns, no significant difference.

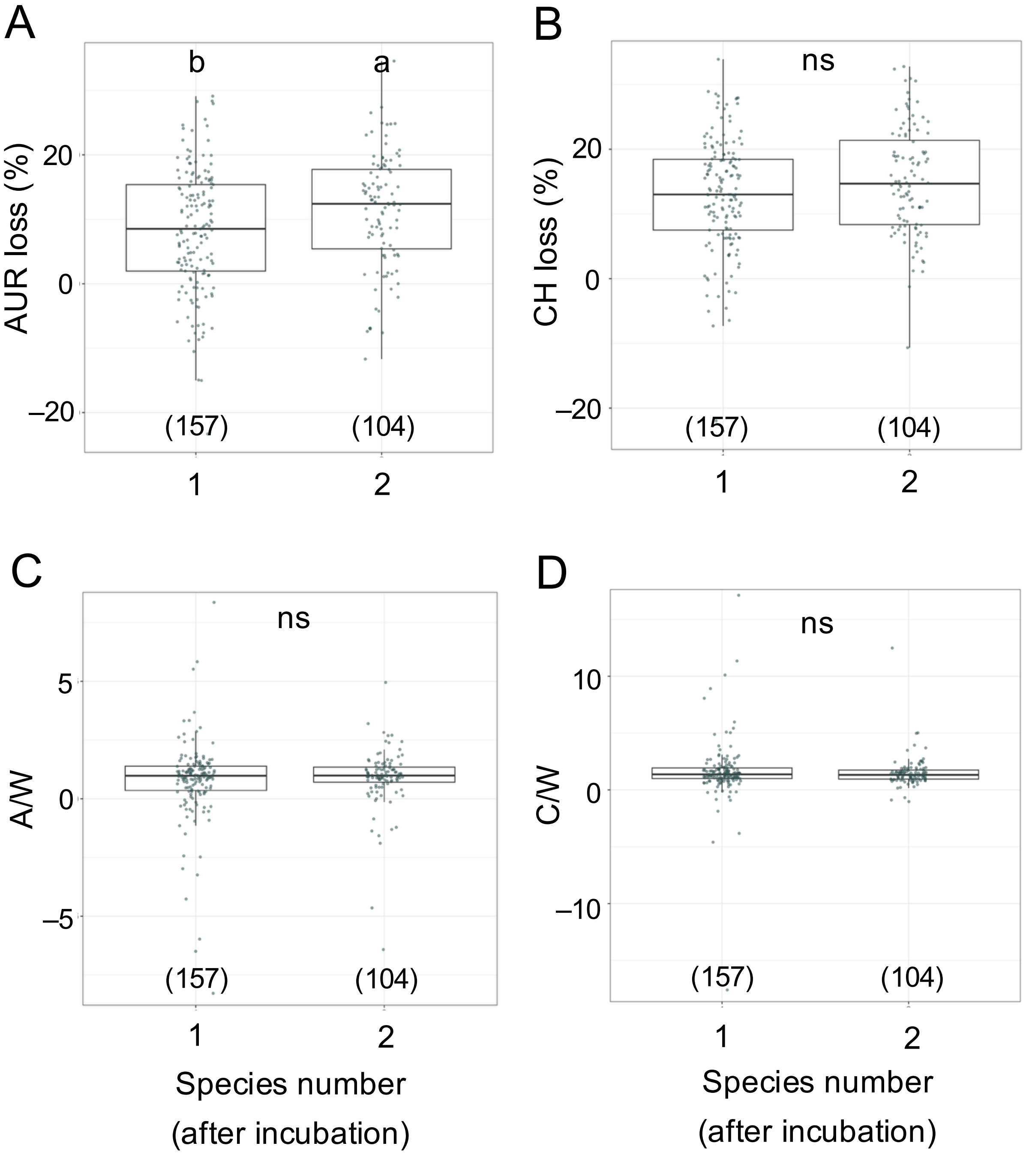

Fig. S6 Effects of fungal species number after the incubation on *Og* blocks' weight loss (%) of AUR (A) and carbohydrate (B), and relative weight loss of AUR (C) and carbohydrate (D) against weight loss of wood. Data of 261 *Og* blocks without replacement and zone lines were used. Four outliers in each A/W and C/W were out of the plots. The different lowercase letters indicate significant difference across the blocks with different fungal species number (Steel–Dwass test, $p < 0.05$). ns, no significant difference. The numbers enclosed in parentheses are the sample number.
